# Effect of intravenous thrombolysis with alteplase on clinical efficacy, inflammatory factors, and neurological function in patients with acute cerebral infarction

**DOI:** 10.1590/1414-431X202010000

**Published:** 2021-03-15

**Authors:** Jinhua Wang, Xia Fang, Dongliang Wang, Yuan Xiao

**Affiliations:** 1 Department of Neurology, The People's Hospital of Beilun District, Beilun Branch Hospital of The First Affiliated Hospital, Zhejiang University School of Medicine, Ningbo, Zhejiang Province, China; 2 Department of Gynecology, The People's Hospital of Beilun District, Beilun Branch Hospital of The First Affiliated Hospital, Zhejiang University School of Medicine, Ningbo, Zhejiang Province, China

## Abstract

This study aimed to explore the effect of intravenous thrombolysis with alteplase on clinical efficacy, inflammatory factors, and neurological function in patients with acute cerebral infarction. A total of 120 patients with acute cerebral infarction were divided into two groups by the random number table method, with 60 patients in each group: observation group (intravenous thrombolysis with alteplase) and control group (intravenous thrombolysis with batroxobin). The clinical efficacy after a 14-day treatment was observed. Serum C-reactive protein (CRP), tumor necrosis factor α (TNF-α), interleukin-6 (IL-6), CD62p, GMP-140, and neuron-specific enolase (NSE) were measured. Scores of National Institutes of Health Stroke Scale (NIHSS), Mini-Mental State Examination (MMSE), and Montreal Cognitive Assessment (MoCA) were determined. The total effective rate in the observation group was 81.67%, which was higher than the 61.67% in the control group (P<0.05). The improvement of inflammatory factors (CRP, TNF-α, IL-6, CD62p, GMP-140, and NSE), NIHSS, MMSE, and MoCA in the observation group was superior to that in the control group (all P<0.05). The modified Rankin scale at three months after hospital discharge in the observation group was lower than that in the control group (P<0.01). Intravenous thrombolysis with alteplase for acute cerebral infarction can enhance the clinical efficacy, alleviate inflammatory response and brain injury, and improve cognitive function, which is worthy of further clinical application and study.

## Introduction

Acute cerebral infarction (ACI) is caused by the stenosis or occlusion of the artery feeding the brain, inducing insufficient cerebral blood supply, which leads to cerebral ischemia and hypoxia and causes brain injury and necrosis, eventually damaging brain function (1,2). The incidence of ACI rises year by year (3), and 30% of patients are the elderly (4–6). ACI is the most common cause of death in China (7). Early thrombolytic therapy for ACI may reduce the degree of injury to the brain (8). Studies have proven the thrombolysis time window for ACI is within 4.5 h (9,10). The thrombolytic drug batroxobin was recommended by the 2014 Guidelines for Diagnosis and Treatment of Acute Ischemic Stroke in China, which has been widely applied in early ACI patients in China with remarkable clinical efficacy (11). In recent years, alteplase, the second generation of thrombolytic drugs, has been applied in clinical practice and is recommended for the treatment of ACI according to evidence-based medicine in Europe and North America. Previous studies found that ACI patients who were evaluated by the National Institute of Neurological Disorders and Stroke (NINDS) criteria could have significant benefits from alteplase and the administration should be as early as possible (12,13). However, alteplase has not been widely used in China because of its high price. Therefore, the advantages and disadvantages of batroxobin and alteplase in thrombolysis effect have not been determined and compared in China (14).

A study revealed that inflammatory response is one of the important pathologic changes of ACI and plays a vital role in thrombosis and nerve function deficit. Abnormal high levels of inflammatory factors can induce oxidative stress response, causing oxidative stress injury to the brain tissue (15). Neuron-specific enolase (NSE) level rapidly increases after early cell injury and is a sensitive indicator for clinical judgment of cell injury (16).

In this study, we investigated the therapeutic effects of batroxobin and alteplase through a comprehensive evaluation in terms of clinical efficacy, effect on inflammatory factors, and improvement of neurological function, expecting to provide more references for clinical practice.

## Material and Methods

### Clinical data

This study was approved by the Ethics Committee of The People's Hospital of Beilun District, Beilun Branch Hospital of The First Affiliated Hospital of Zhejiang University School of Medicine. A total of 120 patients with ACI were recruited from the Department of Neurology of The People's Hospital of Beilun District, Beilun Branch Hospital from January 2017 to June 2018 and they were divided into two groups by the random number table method, with 60 patients in each group: observation group (intravenous thrombolysis with alteplase) and control group (intravenous thrombolysis with batroxobin). All patients were over 65 years old, and all the patients enrolled in this study or their families signed the informed consent form.

### Inclusion criteria

Patients met the diagnostic criteria for ACI of the 2014 Guidelines for Diagnosis and Treatment of Acute Ischemic Stroke in China (17): on hospital admission, ACI patients were identified by CT scans and MRI was performed whenever an alternative diagnosis was suspected; patients were 65-76 years old; patients were diagnosed with ACI for the first time; the time from onset to admission for intravenous thrombolysis was within 4.5 h; the scores of National Institutes of Health Stroke Scale (NIHSS) were 4-15 at admission (18).

### Exclusion criteria

Exclusion criteria were: patients allergic to alteplase or batroxobin; patients with a medical history of craniocerebral trauma, epilepsy, and cerebrovascular disease; patients who could not cooperate with cognitive function evaluation; patients who had coagulopathy or administration of heparin or oral anticoagulant drugs before treatment; patients with cardiopulmonary insufficiency; patients with malignant tumor; patients who had mental disorder that influenced cognition; patients who were in lactation or gestation periods; patients who had hemorrhage in the digestive tract or urinary tract; patients who could not finish the Mini-Mental State Examination (MMSE) and Montreal Cognitive Assessment (MoCA) within 30 min at admission.

### TOAST classification of stroke

The TOAST classification of stroke according to Classification of Subtype of Acute Ischemic Stroke in 1993 was applied (19): atherothrombosis, stroke of undetermined etiology, cardioembolism, small-vessel occlusion, and stroke of other determined etiology.

### Methods

Patients in the two groups were treated according to the treatment regimen of ACI described in the 2014 Guidelines for Diagnosis and Treatment of Acute Ischemic Stroke in China (17), including electrocardiogram monitoring of vital signs, oxygen inhalation, monitoring and controlling blood pressure and blood glucose, dehydration for releasing intracranial pressure, antiplatelet and anticoagulation therapy, the use of free-radical scavenger such as edaravone and other neuroprotective agents, and others. After vital signs were stable, the patients were guided to perform functional rehabilitation.

Patients in the observation group were given alteplase (Boehringer Ingelheim, Germany) at a total dosage of 0.9 mg/kg, in addition to general treatment. Ten percent of the total dosage was injected intravenously within 1 min, and the remaining 90% was dripped intravenously over 1 h. The course of treatment lasted 14 days.

Patients in the control group were given batroxobin (Beijing Tobishi Pharmaceutical Co., Ltd., China), in addition to general treatment. The initial dose on the first day of admission was 10 batroxobin units (BU), which were dripped intravenously over 1 h, and on the third day, 5 BU batroxobin was dripped intravenously over 1 h. The course of treatment lasted 14 days.

### Outcome measures

Primary outcome measures were: treatment efficacy, levels of inflammatory factors, and neurological impairment evaluation. Secondary outcome measures were: cognitive assessment, prognostic assessment, and adverse events.

Treatment efficacy after the 14-day treatment was evaluated according to the ACI criteria described in the 2014 Guidelines for Diagnosis and Treatment of Acute Ischemic Stroke in China (17): the efficacy was evaluated based on the degree of decline of NIHSS score before and after treatment. A decline of 91-100% indicated recovery; a decline of 46-90% indicated remarkable improvement; a decline of 18-45% indicated improvement; a decline of ≤17% indicated no change. Total effectiveness rate was calculated as (number of recovery cases + the number of remarkable improvement cases + the number of improvement cases)/total case number × 100%. The degree of decline was calculated as (NIHSS score before treatment - NIHSS score after treatment)/NIHSS score before treatment × 100%.

Venous blood (5 mL) was drawn at admission and at 8:00 am after the 14-day treatment to evaluate the levels of inflammatory factors of patients. The blood sample was stored in a refrigerator at 4°C for 15 min. The serum and plasma were separated by centrifugation at 3,100 *g* for 10 min (45°C). The plasma was collected and 40 μL phosphate buffered saline containing protease inhibitor was added and this was then stored in a refrigerator at -80°C. Serum C-reactive protein (CRP), tumor necrosis factor α (TNF-α), interleukin-6 (IL-6), CD62p, GMP-140, and neuron-specific enolase (NSE) were measured by an enzyme-linked immunosorbent assay with an automatic microplate reader (Thermo, USA).

Neurologic impairment before and after treatment was accessed using NIHSS scale with a score ranging from 0 to 42. High scores indicate severe neurologic impairment: 0-1: normal; 2-4: mild impairment; 5-15: moderate impairment; 16-20, moderate to severe impairment; over 21, severe impairment (18).

Cognitive function before and after treatment was evaluated using MMSE and MoCA. MMSE includes 19 items with a total score of 30. MoCA has a total score of 30. Low scores indicate worst cognitive function (20).

Prognosis of all patients was evaluated at 1, 2, and 3 months after hospital discharge using the modified Rankin scale by the same physician, who was blinded to their treatment: 0 indicated that the patient was fully recovered; 1, the patient showed symptoms but no significant dysfunction and was able to carry out daily activities; 2, the patient was mildly disabled without the need of assistance but with limited activities; 3, the patient was moderately disabled with the need of assistance for activities but was able to walk without the need of assistance; 4, the patient was moderately disabled without the ability of independent walking and needed assistance from others to meet their life demands; 5, the patient was severely disabled with the need of continuous care and assistance and suffered paralysis, incontinence, etc.; and 6, the patient died. Patients with a Rankin scale score of 2 or below had a good prognosis and those with 3 and above had a poor prognosis.

Adverse reactions, such as cerebral hemorrhage, hematuria, skin allergy, nausea, and vomiting, during treatment were recorded (12).

### Statistical analysis

SPSS 17.0 (IBM, USA) statistical software was used. Continuous variables are reported as means±SD. The data that conformed to normal distribution and homogeneity of variance were analyzed by the *t-*test; the independent-sample *t*-test was used for comparison among groups and the paired-sample *t*-test was employed for comparison before and after treatment within a group. The data that did not confirm to normal distribution and homogeneity of variance are reported as medians and quartiles and analyzed by the rank-sum test. Enumeration data were analyzed by Pearson chi-squared test. P<0.05 indicated a statistically significant difference.

## Results

### General data analysis

There were no significant differences in gender, age, education background, body mass index, time from onset to admission, onset-to-treatment time, the TOAST classification, lesion location, and complications between the two groups (all P>0.05, Table 1).

**Table 1:** Comparison of general data and baseline data of patients with cute cerebral infarction treated with alteplase (observation group) or batroxobin (control group).

	Observation group (n=60)	Control group (n=60)	χ^2^/*t*	P
Gender (male:female)	34:26	28:32	1.201	0.273
Age (years)	68.5±7.1	67.4±5.9	0.692	0.492
Education background (years)	12.50±3.85	11.87±3.97	0.627	0.533
BMI (kg/m^2^)	25.71±3.76	25.59±4.28	0.110	0.913
Time from onset to admission (h)	3.12±0.76	3.21±0.66	0.234	0.786
Onset-to-treatment time (h)	3.78±0.87	3.86±0.79	0.256	0.756
Lesion location			1.382	0.710
Brainstem	7	5		
Lobe	13	10		
Cerebellum	6	9		
Basal ganglia region	34	36		
TOAST classification			0.651	0.957
Atherothrombosis	38	36		
Stroke of undetermined etiology	11	12		
Cardioembolism	6	7		
Small-vessel occlusion	4	3		
Stroke of other determined etiology	1	2		
Hyperlipidemia	34	32	0.135	0.714
Hypertension	40	34	1.269	0.260
Coronary heart disease	20	24	0.574	0.449
Obesity	20	26	1.269	0.260
Hyperhomocysteinemia	44	48	0.745	0.388
Hyperuricemia	38	44	1.386	0.239

Data are reported as means±SD or number. BMI: body mass index.

### Efficacy of treatments

There were significant differences in the number of patients with recovery, remarkable improvement, improvement, and no change between the two groups (all P<0.05). The total effectiveness rate in the observation group (81.67%) was significantly higher than that (61.67%) in the control group (P<0.05, Table 2).

**Table 2:** Comparison of efficacy based on the National Institutes of Health Stroke Scale of patients with acute cerebral infarction treated with alteplase (observation group) or batroxobin (control group).

Group	Recovery	Remarkable improvement	Improvement	No change	Total effective rate
Observation (n=60)	6 (10.00)	28 (46.67)	15 (25.00)	11 (18.33)	49 (81.67)
Control (n=60)	3 (5.00)	17 (28.33)	17 (28.33)	23 (38.33)	37 (61.67)
χ^2^	8.049				5.910
P	0.045				0.015

Data are reported as number (%).

### Inflammatory factors and NSE after treatment

There was no significant difference in inflammatory factors CRP, TNF-α, IL-6, CD62p, GMP-140, and NSE before treatment between the two groups (all P>0.05). These indicators in the two groups were significantly lower after treatment compared with those before treatment (all P<0.05), and the degree of decline in the observation group was higher than that in the control group (P<0.001, Table 3).

**Table 3:** Comparison of inflammatory factors and NSE before and after treatment of patients with cute cerebral infarction treated with alteplase (observation group) or batroxobin (control group).

	Before treatment				After treatment			
	Observation group	Control group	*t*	P	Observation group	Control group	*t*	P
CRP (mg/L)	6.64±1.81	6.90±1.84	0.917	0.363	4.80±1.52^#^	5.57±1.48^#^	4.153	<0.001
TNF-α (μg/mL)	124.51±18.22	123.80±17.87	0.154	0.878	85.01±15.90^#^	102.66±16.12^#^	4.269	<0.001
IL-6 (μg/mL)	351.37±37.86	351.00±37.32	0.038	0.969	273.27±25.64^#^	318.34±29.73^#^	6.287	<0.001
CD62p (pg/mL)	9.11±1.53	9.14±1.59	0.234	0.786	3.17±0.57^#^	5.01±0.68^#^	4.890	<0.001
GMP-140 (ng/mL)	50.89±10.12	50.98±10.01	0.192	0.896	27.65±6.52^#^	36.79±7.61^#^	6.902	<0.001
NSE (μg/L)	36.79±5.92	37.32±5.76	0.352	0.675	15.32±2.65^#^	19.42±3.22^#^	5.898	<0.001

### NIHSS score after treatment

There was no significant difference in the NIHSS score before treatment between the control group (9.70±3.68) and the observation group (9.64±3.62, P>0.05). Compared with before treatment, the NIHSS scores in the control group (5.57±1.48) and the observation group (4.80±1.52) decreased after treatment (P<0.05), however, the degree of decrease in the observation group was higher than that in the control group (P<0.001).

### MMSE and MoCA scores after treatment

**Figure 1:**
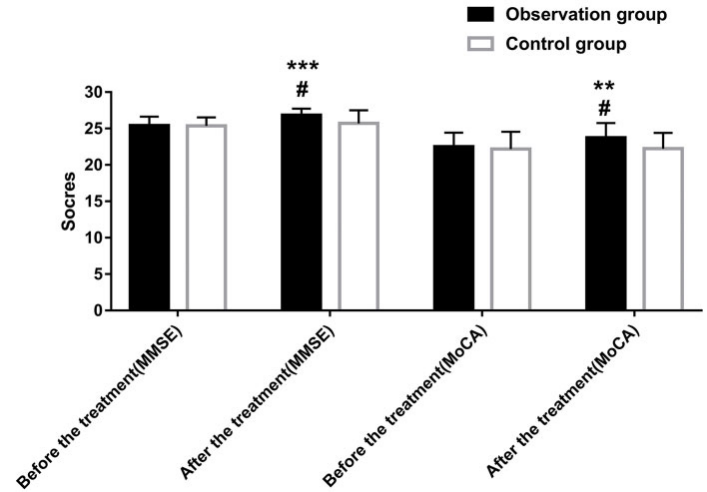
Comparison of cognitive scores after treatment. Data are reported as means±SD. **P<0.01, ***P<0.001 compared with the control group; ^#^P<0.05 compared with before treatment (independent-sample *t*-test and the paired-sample *t*-test). MMSE: mini-mental state examination; MoCA: Montreal Cognitive Assessment.

There was no significant difference in the MMSE and MoCA before treatment between the two groups (both P>0.05). The MMSE and MoCA were significantly improved after treatment compared to those before treatment (both P<0.05). The MMSE and MoCA scores in the observation group were significantly higher than those in the control group after treatment (both P<0.05, Table 4 and Figure 1).

**Table 4:** Comparison of cognitive scores before and after treatment of patients with acute cerebral infarction treated with alteplase (observation group) or batroxobin (control group).

	Before treatment				After treatment			
	Observation group	Control group	*t*	P	Observation group	Control group	*t*	P
MMSE	25.42±1.22	25.37±1.16	0.108	0.914	27.83±0.91^#^	25.70±1.80	5.201	<0.001
MoCA	22.50±1.94	22.20±2.35	0.538	0.592	23.73±2.03^#^	22.23±2.18	2.759	0.008

Data are reported as means±SD. ^#^P<0.05 before and after treatment compared within group. MMSE: mini-mental state examination; MoCA: Montreal Cognitive Assessment.

### Modified Rankin scale scores 3 months after treatment

There was no significant difference in modified Rankin scale scores 1 and 2 months after hospital discharge between the two groups (P>0.05). The modified Rankin scale score 3 months after hospital discharge was significantly lower in the observation group than in the control group (P<0.01). Three months after discharge, the modified Rankin scale scores in the two groups were lower than those at 1 month; moreover, the percentage of patients who had 0-1 scores in the observation group was significantly higher than those in the control group (P<0.05, Table 5).

**Table 5:** Comparison of modified Rankin scale scores of patients with acute cerebral infarction treated with alteplase (observation group) or batroxobin (control group).

	Observation group	Control group	χ^2^ */t*	P
At 1 month after discharge	2.7±0.5	2.9±0.7	0.895	0.126
Rate of patients with scores 0-1 at 1 month after discharge	11 (18.33)	8 (13.33)	0.563	0.453
At 2 months after discharge	2.3±0.5	2.7±0.7	0.912	0.118
Rate of patients with scores 0-1 at 2 months after discharge	16 (26.67)	10 (16.67)	1.768	0.184
At 3 months after discharge	1.9±0.3**	2.5±0.7*	2.796	0.007
Rate of patients with scores 0-1 at 3 months after discharge	25 (41.67)	14 (23.33)	4.596	0.032

Data are reported as means±SD or number and (%). *P<0.05, **P<0.01 compared with the modified Rankin scale score at 1 month after discharge.

### Adverse reactions

No patient died during treatment in the two groups. In the observation group, there was 1 patient with asymptomatic hemorrhagic transformation (evaluated by NINDS criteria), 1 patient with hematuria, and 1 patient with nausea and vomiting, with a total incidence rate of adverse reactions of 5% (3/60). In the control group, there were 2 patients with asymptomatic hemorrhagic transformation (evaluated by NINDS criteria) and 2 patients with nausea and vomiting, with a total incidence rate of adverse reactions of 6.67% (4/60). There was no significant difference in the total adverse reaction incidence rate between the two groups (χ^2^=0.154, P=0.695).

## Discussion

In the early stage of ACI, thrombolytic therapy within the time window of 4.5 h has positive and important significance for prognosis (21). Early thrombolytic therapy can remove vascular blockage as soon as possible, which is beneficial for the reduction of cerebral infarction area and the reconstruction of ischemic circulation to save the ischemic penumbral area (9). Batroxobin, an anti-fibrinogen agent, has the pharmacological effect of reducing blood viscosity, decomposing fibrinogen, inducing thrombolysis and inhibiting thrombosis (22). A large amount of evidence proves that batroxobin is safe and effective in treating ACI, and it is recommended by the 2014 Guidelines for Diagnosis and Treatment of Acute Ischemic Stroke in China (17,23).

Alteplase is a new type of thrombolytic agent, which belongs to gene recombinant thrombolytic drug line. The drug can combine with fibrin after it enters the blood, then activates fibrinogen and promotes the degradation of plasma fibrinogen, thus promoting a thrombolytic effect, and the vessel is rapidly recanalized. Alteplase has become the first choice for the treatment of ACI in European and American countries (10,24,25). In our study, patients with alteplase treatment had a higher decrease in the NIHSS score than those with batroxobin treatment, which is consistent with the results from previous studies (12,13), indicating that patients could obtain more benefits from alteplase than batroxobin.

A Chinese study has shown that thrombolysis with 0.9 mg/kg alteplase has significant advantages and does not increase the risk of intracranial hemorrhage (26). In this study, we found that among ACI patients, total effective rate of alteplase was 81.67%, which was significantly higher than that of batroxobin (61.67%), and no difference was found in the incidence of adverse reaction (hemorrhage) between the two drugs, which was consistent with the above study.

Inflammatory factors play an important role in the development of ACI. CRP is a commonly used clinical indicator, which is synthesized in the liver under the mediation of inflammatory factors like IL-6 (27). Previous study has shown that CRP can reflect the degree of neurological impairment in ACI patients (28). TNF-α has dual biological effects. It not only has an anti-infection effect when its concentration is low in the human body, but also plays a role in the regulation of tissue repair of inflammatory responses. The body's immunity is destroyed when the concentration of TNF-α increases, which could lead to the activation of neutrophils, increased phagocytosis of white blood cells, and the promotion of inflammatory factors secretion, thereby increasing vascular permeability (29). In addition, TNF-α plays an important role in the development and progression of ACI, and its content *in vivo* is positively correlated with cerebral infarction volume and nerve function injury (30). TNF-α can also promote thrombosis and inhibit fibrinolysis (31). IL-6 starts to increase when the brain tissue is damaged, and an appropriate increase of IL-6 is conducive to the functioning of the brain tissue, while an excessive increase will cause further brain tissue damage (32). Previous research has found that alteplase can inhibit the inflammatory response lowering the incidence of hemorrhagic transformation and reducing the risk of hemorrhage after thrombolysis (33). CD62P is a key factor reflecting platelet activation, and GMP140 mediates platelet activation in vascular endothelial injury and promotes the progression of cerebral infarction (34). NSE is the specific key enzyme of glycolysis, which can peak in serum and cerebrospinal fluid 8 h after brain tissue injury (35). The blood-brain barrier permeability changes due to brain tissue damage in brain diseases, and NSE can enter the blood, thus significantly increasing its content in the blood (36). In this study, alteplase had better effects on reducing inflammatory factors and relieving nerve injury than batroxobin, which was consistent with the above results.

Thrombolysis with alteplase can significantly alleviate the hypoxia of brain tissue and reduce the damage of neurogliocytes and neurons, reducing the damage of brain tissue (37). MMSE and MoCA are common evaluation tools to measure the cognitive function of patients (18,20). In this study, alteplase had better effects on reducing brain injury and improving cognitive function than batroxobin, which was consistent with the above research results. Rankin scale is an important indicator to evaluate the prognosis of ACI (38). We found that the score of the modified Rankin scale was lower in patients treated with alteplase than that in those treated with batroxobin 3 months after treatment, suggesting that alteplase could improve the prognosis of ACI patients.

However, the sample size of this study was small, which needs to be further expanded. Additionally, the observing time was short, so the follow-up time needs to be further lengthened. Patients with contraindication for thrombolysis who could not tolerate thrombolytic therapy were not enrolled in this study for control observation. Therefore, these patients should be included in future controlled observational studies to determine the effect of thrombolytic therapy in improving prognosis.

In conclusion, intravenous thrombolytic therapy with alteplase for ACI enhanced clinical improvement, reduced inflammatory response and brain injury, and improved cognitive function, which is worthy of further clinical application and research.
